# Brain-Targeted Epigenome-Wide Association Study of Childhood Internalizing and Externalizing Behaviors: Evidence from a Monozygotic Twin Cohort

**DOI:** 10.21203/rs.3.rs-11156962/v1

**Published:** 2026-10-06

**Authors:** Moira Semple, Taena Hanson, Dominique Hughes, Mary Davis, Kathryn Lemery Chalfant, Candace Lewis

**Affiliations:** Arizona State University; Arizona State University; Arizona State University; Arizona State University; Arizona State University; Arizona State University

**Keywords:** DNA methylation, pediatric, adolescent, internalizing, externalizing, twin design, MZ twin difference analyses

## Abstract

**Background:**

Identifying biologically meaningful epigenetic signatures of childhood internalizing and externalizing behaviors has been challenging because conventional epigenome-wide association studies are limited by small effect sizes, genetic confounding, and uncertain tissue relevance. We therefore combined a brain-targeted epigenome-wide association study (BEWAS) with a monozygotic twin difference design to improve biological specificity while isolating environmentally driven DNA methylation patterns.

**Methods:**

Buccal-derived DNA methylation was analyzed in 415 children from the Arizona Twin Project, including 102 monozygotic twin pairs. DNA methylation analyses were restricted to genes with elevated brain expression or established roles in neurotransmission. Linear mixed-effects models were conducted in the full sample, followed by within-pair MZ difference analyses. Nominally associated CpG sites underwent pathway enrichment analyses, and gene-level findings were compared with previous pediatric DNA methylation studies.

**Results:**

Although no individual CpG sites survived epigenome-wide correction, nominally associated loci converged on robust neurodevelopmental pathways involved in neurogenesis, neuronal differentiation, synaptic organization, and neuronal projection development. Internalizing behaviors demonstrated pathway enrichment in both the population-based and MZ analyses, whereas externalizing enrichment emerged only in the MZ analyses despite the substantially smaller sample size. Gene-level overlap with previous pediatric methylation studies was greater in the MZ analyses, with recurrent associations observed for neurodevelopmental genes including *PTPRN2*, *SHANK2*, *CACNA1C*, and *ADCY8*. Overall, convergence emerged primarily at the level of genes and biological pathways rather than individual CpG sites.

**Conclusions:**

These findings suggest that childhood internalizing and externalizing behaviors are characterized by distributed epigenetic regulation across neurodevelopmental systems. More broadly, they demonstrate how biologically informed epigenomic restriction combined with genetically informative study designs can improve detection and interpretation of molecular signatures relevant to developmental psychopathology, providing a foundation for future biomarker discovery and precision mental health approaches.

## Background

Childhood internalizing and externalizing behaviors represent early-emerging risk factors for later psychiatric disorders (1–4) and functional impairment (5). Although these domains frequently co-occur and predict similar adverse outcomes in adulthood (6), they exhibit substantial heterogeneity in onset, stability, and developmental course (7, 8). Such variability reflects core principles of developmental psychology, including equifinality and multifinality (9), and highlights the need to identify biological mechanisms linking early experiences to divergent behavioral trajectories.

Epigenetic processes, particularly DNA methylation, provide a potential molecular pathway through which environmental exposures become biologically embedded (10, 11), leading to subsequent changes in behavioral and health outcomes (12, 13). DNA methylation is sensitive to social and contextual influences and can regulate gene expression without altering DNA sequence (14), making it a compelling candidate mechanism for experience-dependent neurodevelopment and mental health (15). Pediatric epigenome-wide association studies have reported associations between DNA methylation and internalizing and externalizing behaviors (16–20); however, findings have been inconsistent, likely due to modest effect sizes, genetic confounding, and developmental heterogeneity.

A major limitation of conventional epigenetic studies is their inability to distinguish environmentally driven methylation differences from underlying genetic influences (21). Because DNA methylation is partially heritable and a substantial proportion of CpG variation is influenced by genetic factors, including methylation quantitative trait loci (meQTLs) (22–24), associations observed in unrelated individuals may reflect genetic influences rather than exposure-related biological change. Monozygotic (MZ) twin difference designs directly address this challenge by comparing genetically identical individuals raised in the same family context (25). By accounting for genetic and shared familial influences, MZ twin analyses isolate DNA methylation differences associated with behavioral discordance that are more likely to reflect non-shared environmental experiences. In parallel, tissue relevance remains a critical consideration for psychiatric epigenetics. Because internalizing and externalizing behaviors are inherently driven by neural circuitry (26), restricting analyses to brain-relevant genes may enhance biological specificity and improve signal detection, particularly in pediatric samples where effects are expected to be subtle (27). Additionally, peripheral DNA methylation is associated with variation in brain structure and cognitive function (28–30), supporting its utility as a surrogate tissue for studying neurodevelopment.

The present study integrates these approaches by combining a brain-targeted epigenome-wide association strategy with a genetically informative MZ-twin design to examine DNA methylation associations of childhood internalizing and externalizing behaviors. Using buccal-derived DNA methylation from a pediatric twin cohort, we first conducted a brain-targeted epigenome-wide association study (BEWAS) in the full sample, followed by within-pair difference analyses in an MZ subsample to isolate non-shared environmental influences independent of genetic and shared familial factors. Comparing these complementary analyses enables us to distinguish DNA methylation associations observed at the population level from those that persist after accounting for genetic and shared familial influences. This study extends our previous candidate gene investigations of environmentally associated DNA methylation in stress-responsive and immune pathways by testing these questions at a brain-targeted epigenome-wide scale (30–32). We hypothesized that DNA methylation of brain-targeted genes would be associated with both internalizing and externalizing behaviors. We further hypothesized that MZ twin analyses would distinguish DNA methylation associations associated with behavioral discordance from those reflecting genetic and shared familial influences. By integrating developmental psychopathology theory with molecular and genetically informed methods, this study aims to clarify how early experiences become epigenetically associated and contribute to divergent behavioral trajectories in childhood.

## Methods

### Sample

Participants were recruited from the Arizona Twin Project, a longitudinal cohort designed to investigate the interplay of genetics and environment underlying childhood mental and physical health problems (33, 34). For this study, the full sample was limited to participants whose days between biospecimen collection and behavioral assessment was less than 365 days. Socioeconomic status (SES) was assessed using a composite measure derived from household income-to-needs ratio, primary caregiver years of education, and secondary caregiver years of education. The Zygosity Questionnaire for Young Twins (35), which yields over 95% agreement with genotyping-determined zygosity measures (36, 37), was used to determine twin pair zygosity. Difficult cases were validated with infant birth medical records and photographs taken at home visits, additionally genotyping was conducted on 3% of the most difficult cases (38). All study procedures were approved by the university’s Institutional Review Board, and written informed consent was obtained from primary caregivers, and age-appropriate assent was obtained from twins.

### Saliva Collection and DNA Methylation Arrays

Buccal samples were collected from participants using Mawi iSWAB DNA collection tubes (Mawi DNA Technologies LLC, Hayward, CA) during home visits or parent-collected and mailed to the lab. DNA was extracted from samples with a standard isolation kit (DNA Genotek’s PT-L2P-5) or with the KingFisher Apex machine (ThermoFisher Scientific, Waltham, MA, USA) via standard protocols (39). Sample yield and purity were assessed with NanoDrop ND-1000 methods (ThermoScientific, Wilmington, DE). DNA was treated with sodium bisulfite in an EZ-96 DNA Methylation Kit (Zymo Research, Irvine, CA). DNA methylation was quantified using Illumina Infinium BeadChip arrays scanned on an Illumina iScan System (Illumina, San Diego, CA). Samples were processed across three batches: Batch 1 on EPICv1 (850K) arrays in 2016, and Batches 2 and 3 on EPICv2 (930K) arrays in 2024 and 2025, respectively.

### DNA Methylation Preprocessing

Raw IDAT files were preprocessed in R (4.5.1) using the *minfi* package (40). Sex chromosome probes were filtered out, and data underwent standard quality control measures, including quantile normalization, verification of sex concordance, exclusion of low-quality samples (*p* > 0.01), and removal of probes that had fewer than three beads contributing to the probe signal in more than 5% of samples (41, 42). Data were then normalized and annotated based on Illumina annotation files. Epithelial cell proportions were estimated using the Middleton reference panel (43, 44), developed for cell-type deconvolution in pediatric buccal and saliva-derived DNA methylation samples, and the R package EpiDish (Epigenetic Dissection of Intra-Sample Heterogeneity, 3.8) Robust Partial Correlation (RPC) method. The resulting epithelial cell estimations were used as a covariate in all analyses to control for cell-type heterogeneity, and M-values derived from preprocessing were used for all DNA methylation analyses (45).

### BEWAS Epigenome Restriction

To improve statistical power and increase biological relevance to behavior, we performed a brain-targeted-epigenome-wide association study (BEWAS), testing only sites on genes with relatively elevated expression in brain tissue or that are involved in neurotransmission. Publicly available data was used to identify 2,196 genes with elevated expression in brain tissue in comparison to other tissue types (46). A list of 1,055 neurotransmission-related genes was derived from previous literature (47), which sourced genes from KEGG (48), REACTOME (49), and AmiGO (50) by searching for pathways and ontologies that include glutamate, GABA, dopamine, and/or serotonin. These gene lists combined into a list of 2,854 unique genes. Filtering by sites represented on both EPICv1 and EPICv2 arrays resulted in a final list of 87,730 sites annotated to 1,614 unique genes for use in analyses.

### Behavioral Assessments

Behavior scores for the variables of interest were derived from the parent-reported MacArthur Health and Behavior Questionnaire (HBQ) (51). Internalizing scores represent the mean of Depression, General Anxiety, and Separation Anxiety subscales. Externalizing scores represent the mean of Oppositional Defiant, Conduct Problems, Overt Aggression, and Relational Aggression subscales. Each measure had a possible range of 1–3.

### Statistical Analysis

#### Covariate Selection

Weighted linear predictors were calculated per twin as a representative value of DNA methylation solely for use in model comparisons for covariate selection. This approach was used to reduce computational burden and enable efficient comparison of candidate covariate models. DNA methylation scores were derived from an out-of-fold elastic net model (52, 53) trained on DNA methylation to predict behavior (internalizing or externalizing). Hypothesis-driven covariates of age at sample collection, sex, and epithelial cell proportions were included as fixed effects in the base model predicting behavior, as well as Family ID (identical across twins in a twin pair) as a random intercept. To determine if batch variables significantly contributed to model fit, we conducted principal components analysis (PCA) on the top 100 most variable CpG sites and examined the first eight principal components (PCs) using omnibus MANOVA and per-PC ANOVAs to assess evidence of technical clustering. This base model was compared to models including zygosity, SES, and days between sample collection and behavior assessment as exploratory covariates. Best model fit was determined via consideration of Akaike Information Criterion (AIC) and adjusted R^2^ and was then used as the model for mixed model linear regressions. Identical covariate selection methods were applied to the difference score analyses, with a base model including age at sample collection, sex, and epithelial cell proportion difference scores, compared to models additionally including SES and days between sample collection and behavioral assessment. DNA methylation predictors were recalculated using MZ twin difference scores.

#### Linear Mixed-Effects Models and Within-Pair Difference Linear Models

Linear mixed-effects models (LMMs) were used in the full sample to test associations between DNA methylation at each CpG site and internalizing or externalizing scores. Models included fixed effects for selected covariates (see Results) and a random intercept for twin pair (Family ID) to account for within-family non-independence. To isolate non-shared environmental influences independent of genetic and shared familial factors, MZ within-pair analyses were conducted using difference scores (twin A – twin B). For these analyses, standard linear regression models were applied to test associations between within-pair differences in DNA methylation and corresponding differences in behavioral outcomes, with each twin pair contributing a single observation. Because the unit of analysis was the twin pair, random effects were not required.

#### Over-Representation Analysis

CpG sites passing nominal significance threshold (unadjusted *p* < 0.05) were used for over-representation analysis (ORA), reflecting evidence that complex traits are influenced by many small, distributed effects (54, 55). This approach is analogous to polygenic score methodology, where aggregating numerous subthreshold genetic or epigenetic associations often improves phenotype prediction compared with restricting analyses to only genome-wide significant loci. (56–58). Accordingly, ORA was used to determine whether nominally associated CpG sites converged on biologically meaningful pathways despite the absence of epigenome-wide significant individual loci. ORA was conducted using the *gometh* function from the Bioconductor missMethyl package (R version 4.4.1), using all CpG sites passing quality control and the BEWAS filtering specified as the background set. This function accounts for probe-number and multi-gene bias inherent to DNA methylation array data, reducing bias towards genes represented by larger numbers of CpG probes. *Gometh* assesses Gene Ontology (GO: Biological Process, Molecular Function, Cellular Component) and KEGG pathway enrichment analyses, with statistical significance determined using Benjamini–Hochberg false discovery rate (FDR) correction (q < 0.05).

## Results

### Demographics

Demographic characteristics are presented in Table 1. Participants had a mean age of 11.37 ± 2.22 years in the full sample and 10.38 ± 2.31 years in the MZ subsample. The full sample was 50.6% female, 57.11% Non-Hispanic White and 33.25% Hispanic, with similar demographic distributions observed in the MZ subsample.

### Model Comparisons for Covariate Selection – Full Sample

In PCA-based analysis of array variables, batch showed a multivariate effect (Pillai’s trace = 1.06, *p* < 2 × 10^− 16^), with Batch 1 clustering distinctly from Batches 2 and 3, consistent with differences between EPICv1 and EPICv2 arrays. Chip also demonstrated a significant effect (Pillai’s trace = 1.56, *p* < 7.63 × 10^− 6^); however, chip-level clustering was entirely nested within batch, indicating that chip effects reflect array-type differences rather than independent technical structure. Row position showed minimal effects and did not survive FDR correction. Based on these results, batch was included as the array covariate in the base model.

Weighted linear predictors were calculated via elastic net per twin as a representative value of DNAm for use in model comparisons. The base model included age at sample collection, sex, epithelial cell proportions, and batch as fixed effects, and a random intercept for twin pair (Family ID). For internalizing behavior, zygosity, SES, and days between sample collection and behavior assessment substantially worsened model fit relative to the base model (ΔAIC = + 8.88, + 7.99, and + 16.91, respectively). For externalizing behavior, SES produced a ΔAIC value < 2, indicating statistical equivalence to the base model, whereas zygosity and days between samples collection and behavior assessment again worsened fit (ΔAIC = + 11.88 and + 16.76, respectively). Because none of the exploratory covariates consistently improved model fit across outcomes, the final linear mixed-effects model predicting behavior included age at collection, sex, epithelial cell proportions, and batch as fixed effects, and twin pair as a random intercept.

### Model Comparisons for Covariate Selection – MZ-Twin Subsample

Weighted linear predictors were calculated via elastic net per twin pair as a representative value of DNAm difference scores for use in model comparisons. The base model included twin pair age at sample collection, twin pair sex, and epithelial cell proportion difference scores. For internalizing behavior, adding SES or days between did not significantly improve model fit compared to the base model (ΔAIC = + 0.73 and + 0.01, respectively). Externalizing behavior had similar results, with neither SES or days between significantly improving model fit (ΔAIC = + 1.69 and + 1.97, respectively). Because neither of the exploratory covariates consistently improved model fit across outcomes, the final linear model predicting behavior included twin pair age at collection, twin pair sex, and epithelial cell proportion difference scores.

### Linear Mixed-Effects Models and Over-Representation Analysis – Full Sample

A total of 3,470 CpG sites were nominally associated with internalizing behaviors (Supplementary Table 1), of which 17.5% were located within promoter regions and 53.54% resulted in negative regression beta coefficients, indicating lower methylation with higher internalizing scores. Similarly, 3,606 CpG sites were nominally associated with externalizing behaviors (Supplementary Table 2), with 20.13% located within promoter regions and 46.67% resulted in negative regression beta coefficients.

Despite this comparable number of nominal associations across behavioral domains, divergent patterns emerged at the pathway level. Over-representation analysis using *gometh* identified 15 significantly enriched pathways for internalizing behaviors (*q* < 0.05; Table 2; Supplementary Table 3), all within the Gene Ontology Biological Process category, whereas no significant pathway enrichment was observed for externalizing behaviors. Convergent DNAm-associated genes across behavioral domains and analytic frameworks are shown in Fig. 1.

### Linear Models and Over-Representation Analysis - MZ-Twin Difference

Co-twin differences in a total of 4,682 CpG sites were nominally associated with differences in internalizing behaviors (Supplementary Table 4), of which 17.34% were located within promoter regions and 47% resulted in positive regression coefficients, indicating methylation differences in twin pairs are associated with differences in internalizing behaviors. Similarly, twin differences in 4,096 CpG sites were nominally associated with differences in externalizing behaviors (Supplementary Table 5), with 14.96% located within promoter regions and 47% resulted in positive regression coefficients.

Both internalizing and externalizing behaviors demonstrated robust pathway-level enrichment in the MZ subsample. For internalizing behaviors, ORA using *gometh* identified 17 significantly enriched pathways (*q’s* < 0.05; Table 3; Supplementary Table 6), with the majority falling within the Gene Ontology Cellular Component category (70.58%), followed by Biological Processes (17.6%) and Molecular Function (11.76%). For externalizing behaviors, 34 significantly enriched pathways were identified using *gometh* (*q’s* < 0.05; Table 4; Supplementary Table 7), predominantly within the Gene Ontology Biological Process category (82.35%), with smaller proportions in Molecular Function (8.82%) and Cellular Component (8.82%). Convergent DNAm-associated genes across behavioral domains and analytic frameworks are shown in Fig. 1.

### Concordance of CpG Effect Sizes Across Analytic Frameworks

To evaluate the consistency of DNA methylation associations across analytic approaches, we compared CpG-level effect sizes estimated from the full sample and monozygotic (MZ) twin difference models (Fig. 2). Effect sizes were moderately correlated for both internalizing (Spearman ρ = 0.52, p < 2.2e-16) and externalizing behaviors (ρ = 0.40, p < 2.2e-16), indicating substantial concordance in the direction and magnitude of methylation-behavior associations across models. However, considerable dispersion around the line of best fit was observed, with numerous CpG sites demonstrating nominal significance in only one analytical framework These differences suggest that while many methylation associations are shared across approaches, others are sensitive to accounting for genetic and shared familial influences. Overall, comparison of the two analytical frameworks identified both overlapping associations and associations unique to each model, highlighting the value of the MZ twin design for distinguishing methylation signals associated with behavioral discordance independent of genetic and shared familial influences.

### Concordance of Gene -Level Effects with Prior Literature

To evaluate the consistency of our findings with the existing literature, we compared gene-level associations with those reported in previous DNA methylation studies of childhood internalizing and externalizing behaviors, depressive symptoms, psychopathology, and early-life adversity (Table 5). Despite substantial differences across studies in cohort characteristics, developmental stage, biospecimen, and analytic approach, multiple previously reported loci overlapped with at least one of the four analytical models. Notably, overlap was considerably more common in the MZ analyses than in the population-based analyses, with recurrent genes identified across independent cohorts. Overall, these findings demonstrate gene-level convergence across pediatric epigenetic studies despite limited replication at individual CpG sites.

## Discussion

Childhood internalizing and externalizing behaviors are early indicators of risk for later psychiatric disorders, yet their molecular correlates remain poorly understood. By combining a brain-targeted epigenome-wide association study with a monozygotic twin difference design, we identified pathway-level DNA methylation signatures associated with both behavioral domains. Although no individual CpG associations survived epigenome-wide correction, nominally associated loci converged on significant neurodevelopmental pathways. Internalizing behaviors showed enrichment in both analytic frameworks, whereas externalizing enrichment emerged only in the MZ analyses.

Previous epigenome-wide association studies of childhood internalizing and externalizing behaviors have generally reported sparse and inconsistent findings, even in large cohorts, with few reproducible CpG-level associations (16–20). Our results were consistent with this literature at the single-CpG level, as associations were modest and did not survive correction for multiple testing. However, nominally associated loci converged on robust pathway-level enrichment, particularly in the MZ analyses. This pattern may reflect the complementary strengths of the present analytic framework. Restricting analyses to genes with elevated brain expression or established roles in neurotransmission reduced the multiple comparison burden while increasing biological specificity, whereas the MZ co-twin design reduced genetic confounding and between-individual heterogeneity, allowing methylation differences due to the non-shared environment to be examined independently of inherited liability. Together, these approaches may improve sensitivity to coordinated biological signals composed of many individually small epigenetic effects (54, 55).

Despite substantial heterogeneity in developmental stage, tissue source, and analytic strategy, previous pediatric methylation studies showed meaningful gene-level convergence with the present findings (Table 5) (16–20). Shared loci mapped predominantly to neurodevelopmental and synaptic processes, including neurogenesis, neuronal differentiation, membrane signaling, and projection development. Convergence also emerged at the pathway level despite the use of different enrichment platforms across studies. For example, previous pediatric studies similarly implicated nervous system development, axon development and guidance, cell communication, signaling pathways, and synaptic organization, with an exact Gene Ontology match for regulation of cell communication observed in both our MZ externalizing analysis and Dunn et al. Although the specific pathway annotations differed in granularity, the broader biological themes were remarkably consistent across studies. Overall, these findings suggest that reproducibility in pediatric behavioral epigenetics may emerge more clearly at the level of genes and biological pathways than at individual CpG sites. The greater convergence observed in the MZ analyses further suggests that controlling for genetic and shared familial influences facilitates detection of these meaningful, yet distributed signals.

The comparison between the full sample and monozygotic (MZ) analyses provided additional insight into the biological architecture of childhood internalizing and externalizing behaviors. CpG-level effect sizes were moderately correlated across analytic frameworks, indicating substantial concordance between population-based and genetically informed within-pair analyses. However, pathway-level enrichment differed markedly between behavioral domains. Internalizing behaviors demonstrated significant enrichment in both the full sample and MZ analyses, suggesting that these biological signals are detectable across complementary analytic frameworks. In contrast, significant pathway enrichment for externalizing behaviors emerged only in the MZ analyses despite the substantially smaller sample size. This pattern suggests that epigenetic variation associated with externalizing behaviors may become more readily detectable after accounting for genetic and shared familial heterogeneity. Consistent with behavioral genetic models emphasizing the importance of non-shared environmental experiences in behavioral divergence among genetically identical individuals (30, 59), these findings raise the possibility that environmentally associated epigenetic signals contributing to externalizing behaviors may be partially masked in conventional population-based analyses.

Across behavioral domains, enriched pathways converged on neuronal maturation, synaptic connectivity, and neural circuit formation. Internalizing findings emphasized nervous system development and neuronal differentiation, whereas the MZ externalizing findings additionally implicated neuronal morphogenesis, signaling, and axonal and dendritic development. These findings support developmental psychopathology models proposing that early environmental experiences become epigenetically embedded (10, 11) and influence neural development and later behavioral trajectories (12, 13).

Notably, *PTPRN2, SHANK2, CACNA1C*, and *ADCY8* repeatedly emerged across behavioral domains and analytic frameworks. *PTPRN2* lies within genomic regions associated with schizophrenia and bipolar disorder and has been implicated in neuronal development and synaptic function (60). Variants in *SHANK2*, a postsynaptic scaffolding protein essential for dendritic spine maturation and excitatory synapse organization, have been consistently associated with autism spectrum disorder and other neurodevelopmental disorders (61–63). Similarly, *CACNA1C*, which encodes the L-type voltage-gated calcium channel CaV1.2 localized in synaptic spines, dendritic shafts, and neuronal cell bodies, is one of the most consistently replicated transdiagnostic genetic risk loci across major psychiatric disorders (64, 65). *ADCY8*, which encodes a calcium/calmodulin-stimulated adenylyl cyclase that couples neuronal calcium influx to intracellular cAMP signaling, regulates long-term potentiation, learning, memory, and stress-responsive synaptic plasticity (66). Increasing evidence from human genetic studies further implicates *ADCY8* in multiple psychiatric disorders, including bipolar disorder, schizophrenia, autism spectrum disorder, obsessive-compulsive disorder, and posttraumatic stress disorder (67). Because *ADCY8* functions immediately downstream of calcium entry through channels such as *CACNA1C*, the repeated identification of both genes suggests convergence on activity-dependent intracellular signaling pathways that regulate synaptic plasticity and experience-dependent neural adaptation. Given that childhood internalizing and externalizing behaviors are well-established precursors of later psychiatric disorders (1–4), these findings raise the possibility that epigenetic variation may contribute to early molecular vulnerability for later psychiatric disorders.

Although early-life adversity was not the focus of the present study, we observed notable overlap with previous epigenetic studies of childhood adversity. For example, DNA methylation of *RHEBL1*, previously associated with maternal psychopathology, and *RNF220*, associated with childhood financial stress, were both associated with internalizing and externalizing in the current study (68). We also observed overlap with genes previously associated with childhood abuse and depression, including *TPPP, GRIN1*, and *BDNF*, all of which were represented within our enriched neurodevelopmental pathways (69). Together, these overlaps identify candidate epigenetic links between childhood adversity and behavioral variation.

Several limitations should be considered. First, although saliva-derived DNA methylation demonstrates greater correspondence with brain tissue than many other peripheral tissues (70–72), peripheral tissues cannot fully recapitulate brain-specific epigenetic processes. Recent work has further demonstrated that peripheral DNA methylation is associated with brain structure, supporting its utility for investigating brain-relevant biology despite tissue-specific differences (28, 29). Second, pathway analyses were intentionally conducted using nominally associated CpG sites to maximize sensitivity for detecting distributed biological signals, consistent with polygenic and polyepigenetic models of complex traits (54, 55) However, because many nominal CpG associations are expected to occur by chance in epigenome-wide analyses, individual CpG findings should be interpreted cautiously. Accordingly, the gene- and pathway-level findings reported here should be considered hypothesis-generating until replicated in independent cohorts. Finally, longitudinal studies incorporating repeated biospecimen collection will be necessary to determine whether these methylation differences precede internalizing and externalizing behavioral development or emerge as consequences of these behaviors.

## Conclusions

In summary, this study demonstrates that integrating biologically informed epigenomic restriction with genetically informative study designs reveals coordinated neurodevelopmental DNA methylation signatures associated with childhood internalizing and externalizing behaviors. Although individual CpG associations remained modest, convergence at the level of genes and biological pathways suggests that childhood behavioral vulnerability is characterized by distributed epigenetic regulation across neurodevelopmental systems. These findings further support the use of peripheral DNA methylation as an accessible and scalable surrogate for brain-relevant biological processes and highlight the value of brain-targeted epigenome-wide approaches for improving biological interpretability. More broadly, identifying the epigenetic pathways through which early experiences become biologically embedded may ultimately facilitate biologically informed patient stratification and the development of interventions targeting the molecular systems underlying behavioral vulnerability. As therapies designed to promote neuroplasticity continue to emerge (73), understanding these epigenetic signatures may also provide mechanistically informed biomarkers for predicting treatment response and monitoring therapeutic change.

## Supplementary Material

Supplementary Files

This is a list of supplementary files associated with this preprint. Click to download.

- Table5.xlsx
- SupplementaryTable6MZIntGenes.xlsx
- SupplementaryTable7MZExtGenes.xlsx
- SupplementaryTable3FullIntGenes.xlsx
- SupplementaryTable2FullExt.csv
- SupplementaryTable4MZInt.csv
- SupplementaryTable5MZExt.csv
- SupplementaryTable1FullInt.csv

Table 5 is available in the Supplementary Files section.

## Figures and Tables

**Figure 1:**
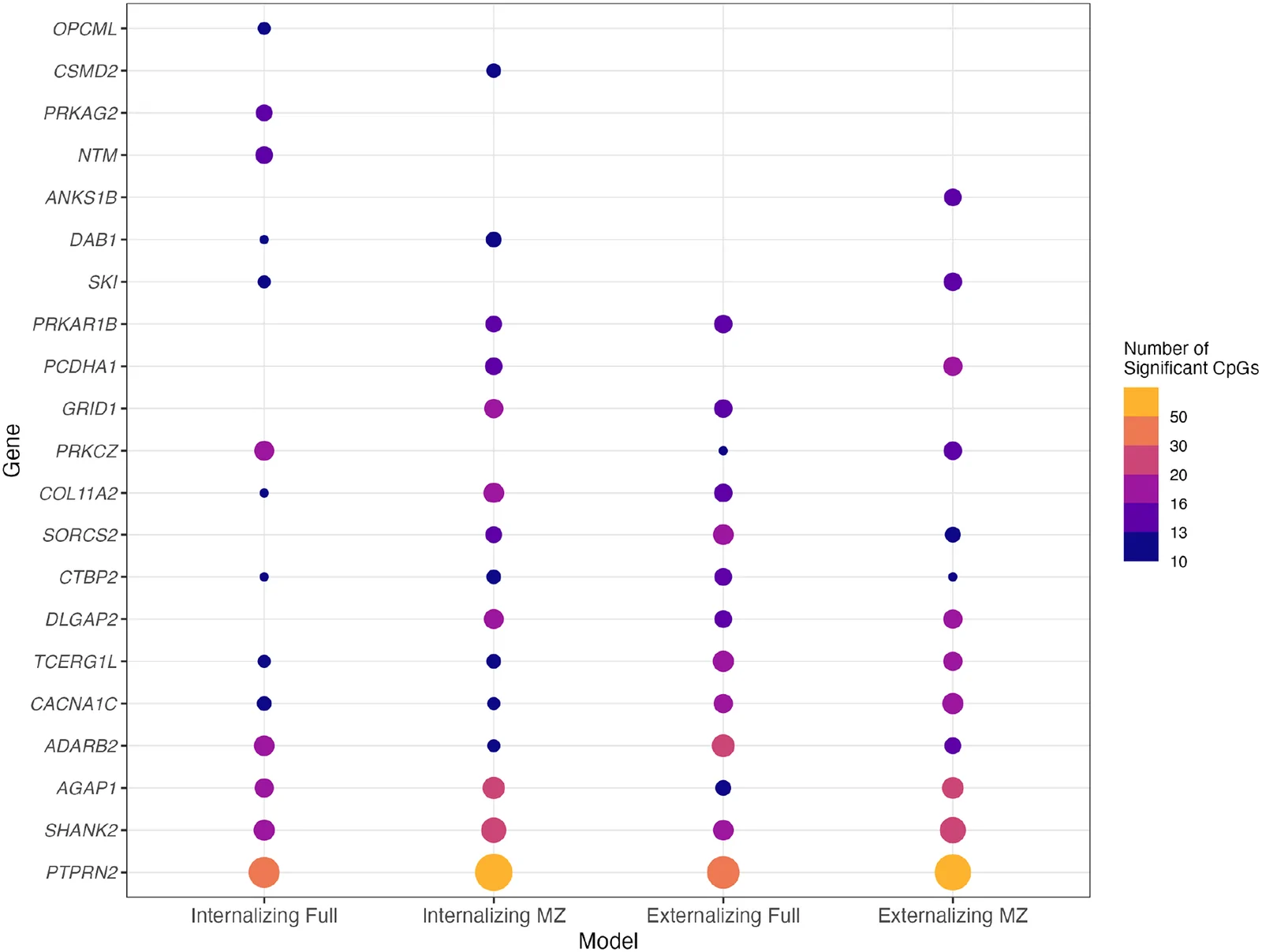
Comparison of top DNA methylation-associated genes across population-based and monozygotic twin models. Genes are ranked according to the number of annotated nominally significant CpG sites (p < 0.05) within each analytic model. The top ten genes from each model are displayed, with overlapping genes shown once. Bubble size and color indicate the number of annotated nominally significant CpG sites for each gene-model combination. Rows represent genes and columns represent analytic models.

**Figure 2:**
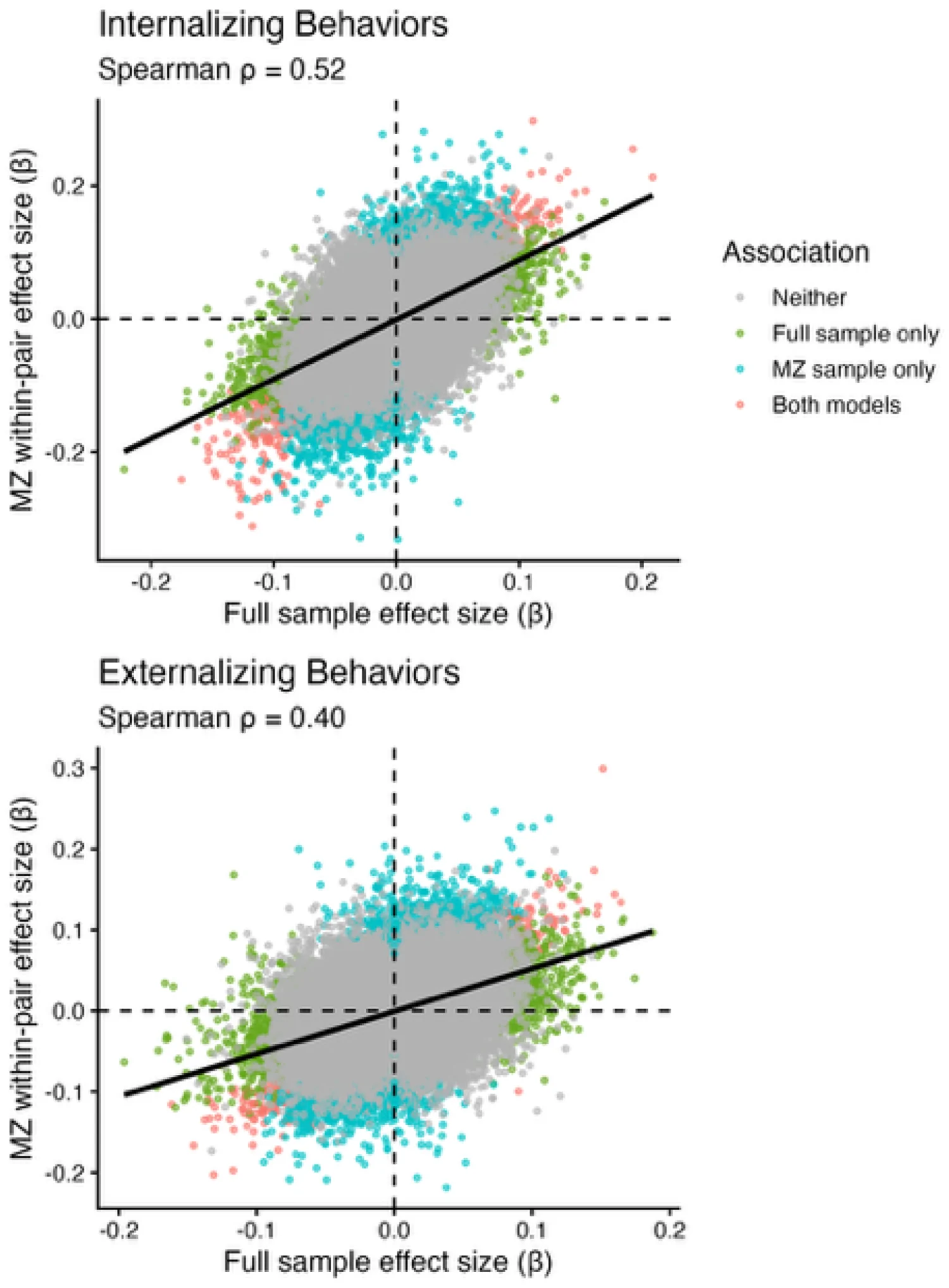
Concordance of CpG effect sizes across analytic frameworks. Scatter plots compare CpG-level effect sizes from the full sample model (x-axis) and monozygotic (MZ) twin difference model (y-axis) for internalizing (left) and externalizing (right) behaviors. Each point represents a CpG site and is colored according to whether it was nominally associated (p < 0.05) in neither model, the full sample model only, the MZ model only, or both models. Solid lines indicate linear regression fits, and dashed lines denote zero effect.

**Table 1:** Sample Demographics

Age (yrs)		Full Sample	MZ Subsample
			
	Mean ± SD	11.37 ± 2.22	10.38 ± 2.31
	Range	7.04–16.33	7.04–16.33
Sex (%)			
	Male	49.40	41.18
	Female	50.60	58.82
Race/Ethnicity (%)			
	Non-Hispanic White	57.11	55.88
	Hispanic	33.25	32.35
	Other	9.64	11.77

**Table 2:** Pathway Enrichment Analysis of CpG sites Associated with Internalizing Behaviors.

GO:ID	Ontology	Term	P	FDR
GO:0007399	BP	nervous system development	1.74E-06	9.48E-03
GO:0030182	BP	neuron differentiation	9.11E-07	9.48E-03
GO:0048699	BP	generation of neurons	2.32E-06	9.48E-03
GO:0022008	BP	neurogenesis	4.86E-06	1.49E-02
GO:0000902	BP	cell morphogenesis	1.48E-05	2.55E-02
GO:0010975	BP	regulation of neuron projection development	1.43E-05	2.55E-02
GO:0031344	BP	regulation of cell projection organization	1.26E-05	2.55E-02
GO:0048666	BP	neuron development	1.87E-05	2.55E-02
GO:0048858	BP	cell projection morphogenesis	1.68E-05	2.55E-02
GO:0120035	BP	regulation of plasma membrane bounded cell projection organization	2.08E-05	2.56E-02
GO:0048731	BP	system development	3.08E-05	3.44E-02
GO:0032989	BP	cellular anatomical entity morphogenesis	4.01E-05	3.51E-02
GO:0048812	BP	neuron projection morphogenesis	3.83E-05	3.51E-02
GO:0120039	BP	plasma membrane bounded cell projection morphogenesis	3.83E-05	3.51E-02
GO:0031175	BP	neuron projection development	4.53E-05	3.71E-02

Note: BP = Biological Process; CC = Cellular Component; MF = Molecular Function; HP = Human Phenotype Ontology. Pathway enrichment was performed using missMethyl package in R version 4.4.1, function gometh() was used to identify enriched pathways. P.DE represents the p-value for over-representation of the GO or KEGG term. False discovery rate was controlled using the Benjamini-Hochberg (BH) adjustment.

**Table 3:** Pathway Enrichment Analysis of CpG sites Associated with Internalizing Behaviors in Monozygotic Subsample (Twin Differences)

GO:ID	Ontology	Term	P	FDR
GO:0045202	CC	synapse	4.30E-08	5.28E-04
GO:0030054	CC	cell junction	1.75E-07	1.08E-03
GO:0043005	CC	neuron projection	1.93E-06	7.90E-03
GO:0006810	BP	transport	7.04E-06	2.16E-02
GO:0005886	CC	plasma membrane	1.52E-05	2.45E-02
GO:0051179	BP	localization	1.22E-05	2.45E-02
GO:0071944	CC	cell periphery	1.60E-05	2.45E-02
GO:0098794	CC	postsynapse	1.28E-05	2.45E-02
GO:0051234	BP	establishment of localization	1.82E-05	2.48E-02
GO:0042995	CC	cell projection	2.75E-05	3.38E-02
GO:0016020	CC	membrane	3.79E-05	4.23E-02
GO:0030424	CC	axon	4.32E-05	4.41E-02
GO:0098793	CC	presynapse	4.94E-05	4.67E-02
GO:0015318	MF	inorganic molecular entity transmembrane transporter activity	6.77E-05	4.89E-02
GO:0030554	MF	adenyl nucleotide binding	5.63E-05	4.89E-02
GO:0097060	CC	synaptic membrane	6.70E-05	4.89E-02
GO:0120025	CC	plasma membrane bounded cell projection	6.03E-05	4.89E-02

Note: BP = Biological Process; CC = Cellular Component; MF = Molecular Function; HP = Human Phenotype Ontology. Pathway enrichment was performed using MissMethyl package in R version 4.4.1, function gometh() was used to identify enriched pathways. P.DE represents the p-value for over-representation of the GO or KEGG term. False discovery rate was controlled using the Benjamini-Hochberg (BH) adjustment.

**Table 4:** Pathway Enrichment Analysis of CpG sites Associated with Externalizing Behaviors in Monozygotic Subsample (Twin Differences)

GO:ID	Ontology	Term	P	FDR
GO:0007399	BP	nervous system development	1.02E-07	1.25E-03
GO:0048731	BP	system development	2.09E-07	1.28E-03
GO:0032501	BP	multicellular organismal process	6.01E-07	2.46E-03
GO:0030182	BP	neuron differentiation	9.87E-07	3.03E-03
GO:0022008	BP	neurogenesis	1.59E-06	3.26E-03
GO:0048699	BP	generation of neurons	1.46E-06	3.26E-03
GO:0032989	BP	cellular anatomical entity morphogenesis	3.46E-06	5.56E-03
GO:0048666	BP	neuron development	4.05E-06	5.56E-03
GO:0048812	BP	neuron projection morphogenesis	4.53E-06	5.56E-03
GO:0120039	BP	plasma membrane bounded cell projection morphogenesis	4.53E-06	5.56E-03
GO:0048667	BP	cell morphogenesis involved in neuron differentiation	5.76E-06	5.89E-03
GO:0048858	BP	cell projection morphogenesis	5.42E-06	5.89E-03
GO:0007275	BP	multicellular organism development	9.37E-06	7.64E-03
GO:0023051	BP	regulation of signaling	8.28E-06	7.64E-03
GO:0032502	BP	developmental process	9.70E-06	7.64E-03
GO:0043167	MF	ion binding	1.03E-05	7.64E-03
GO:0050896	BP	response to stimulus	1.06E-05	7.64E-03
GO:0036094	MF	small molecule binding	1.24E-05	8.44E-03
GO:0030154	BP	cell differentiation	1.53E-05	9.39E-03
GO:0048869	BP	cellular developmental process	1.53E-05	9.39E-03
GO:0010646	BP	regulation of cell communication	1.79E-05	9.56E-03
GO:0043005	CC	neuron projection	1.75E-05	9.56E-03
GO:0048468	BP	cell development	1.70E-05	9.56E-03
GO:0048856	BP	anatomical structure development	1.88E-05	9.61E-03
GO:0060560	BP	developmental growth involved in morphogenesis	1.96E-05	9.62E-03
GO:0031175	BP	neuron projection development	2.34E-05	1.10E-02
GO:0007409	BP	axonogenesis	3.99E-05	1.77E-02
GO:0032879	BP	regulation of localization	4.04E-05	1.77E-02
GO:0005737	CC	cytoplasm	4.44E-05	1.88E-02
GO:0048589	BP	developmental growth	8.05E-05	3.29E-02
GO:0030424	CC	axon	9.46E-05	3.74E-02
GO:0007154	BP	cell communication	1.12E-04	4.26E-02
GO:0046872	MF	metal ion binding	1.15E-04	4.26E-02
GO:0016358	BP	dendrite development	1.24E-04	4.49E-02

Note: BP = Biological Process; CC = Cellular Component; MF = Molecular Function; HP = Human Phenotype Ontology. Pathway enrichment was performed using missMethyl package in R version 4.4.1, the function gometh() was used to identify enriched pathways. P.DE represents the p-value for over-representation of the GO or KEGG term. False discovery rate was controlled using the Benjamini-Hochberg (BH) adjustment.

## Data Availability

The data that support the findings of this study are available from the corresponding author upon reasonable request by qualified researchers.
